# The value of mobile magnetic resonance imaging in early warning for stroke: A prospective case-control study

**DOI:** 10.3389/fnins.2022.975217

**Published:** 2022-08-12

**Authors:** Miaomiao Liu, Qingyang Li, Guoqiang Chen, Ning Su, Maorong Zhou, Xiaolin Liu, Kai Sun

**Affiliations:** ^1^The Third People’s Hospital of Longgang District, Shenzhen, China; ^2^Graduate School of Baotou Medical College, Inner Mongolia University of Science and Technology, Baotou, China; ^3^Department of Radiology, The First Clinical Medical College of Inner Mongolia Medical University, Huhhot, China; ^4^Department of Radiology, Baotou Central Hospital, Baotou, China

**Keywords:** stroke, mobile MRI, early warning model, joint indicators, case-control study

## Abstract

**Aims:**

To evaluate the predictive value of mobile magnetic resonance imaging (MRI) in screening stroke.

**Methods:**

This was a prospective case-control study performed on healthy residents over 40 years old in remote rural areas of northern China between May 2019 and May 2020. Multivariate logistic regression and receiver operator characteristic curve (ROC) analysis were used to evaluate the screening model.

**Results:**

A total of 1,224 patients (500 [40.8%] men) enrolled, including 56 patients who suffered from stroke (aged 64.05 ± 7.27). The individuals who developed stroke were significantly older (*P* < 0.001), had a significantly higher occurrence of heart disease (*P* = 0.015), diabetes (*P* = 0.005), dyslipidemia (*P* = 0.009), and significantly increased waist circumference (*P* = 0.02), systolic blood pressure (SBP) (*P* = 0.003), glycosylated hemoglobin (HbA1c) level (*P* = 0.007), triglyceride (TG) level (*P* = 0.025), low density lipoprotein cholesterol (LDL-c) level (*P* = 0.04), and homocysteine (HCY) level (*P* < 0.001). Multivariate logistic regression analysis showed that age (OR = 1.055, 95% CI: 1.017–1.094, *P* = 0.004), HCY (OR = 1.029, 95% CI: 1.012–1.047, *P* = 0.001) and mobile MRI (OR = 4.539, 95% CI: 1.726–11.939, *P* = 0.002) were independently associated with stroke. The area under the curve (AUC) of the combined model including national screening criteria, mobile MRI results, and stroke risk factors was 0.786 (95% CI: 0.721–0.851), with a sensitivity of 69.6% and specificity of 80.4%.

**Conclusion:**

Mobile MRI can be used as a simple and easy means to screen stroke.

## Introduction

Stroke is the second leading cause of death and disability worldwide (GBD 2019 Stroke Collaborators, 2021). Global burden of disease data show that there are differences in health outcomes among patients residing in different provinces of China, with central and western rural areas facing the worst outcomes (Zhou et al., 2019). China’s rural poverty alleviation and development plan (2010–2020) proposed to achieve equitable access to public health and basic medical services by 2020 (Old Area, 2011). However, the development of health services in rural areas remains a challenge.

Cerebral small vessel disease (cSVD) is a common cause of stroke and encompasses a large class of cerebrovascular diseases involving arterioles, capillaries, and venules. Ischemic stroke and cSVD share similar neuroimaging features, including evidence of recent subcortical small infarction, lacunar infarcts, white matter hyperintensity (WMH), perivascular space enlargement (EPVs), micro-hemorrhage and brain atrophy. In the pathogenesis of sporadic arteriosclerosis associated with cSVD, the total load of cSVD is an important predictor of stroke events, overall cognitive impairment, mental illness, and later quality of life. The previous cSVD scoring system did not measure brain atrophy (Chen et al., 2018). Imaging research reports show that the existence and severity of cSVD is directly correlated to the degree of brain atrophy (Appelman et al., 2009; Aribisala et al., 2012). In addition, the American Heart Association and American Stroke Association list brain atrophy as a symptom of cSVD (Smith et al., 2017). Therefore, in this study, brain atrophy was included in a visual scoring system of MRI results. This system was introduced to represent the total load of cSVD and cerebrovascular diseases and establish a model of stroke risk.

Accurate risk assessment and the early detection of stroke are essential for primary prevention. The Framingham stroke risk assessment scale (FSRAS) was the first simple risk assessment tool proposed abroad and has been widely implemented (Uiterwijk et al., 2018). In addition, there are other early warning models in use, such as the stroke risk assessment app (Gawidan et al., 2021), UK QRISK (Hippisley-Cox et al., 2007) and QRISK2 (Hippisley-Cox et al., 2008), and the predict cardiovascular and cerebrovascular risk assessment model (Guidelines for Primary Prevention of Cerebrovascular Diseases in China, 2019). There is a lack of evidence-based health policies targeting rural populations in China (Wang et al., 2019). Cervical artery ultrasound and transcranial Doppler ultrasound can be used to detect stroke. It can judge the degree and scope of cerebral and cervical vascular stenosis and provide important information for clinical intervention. However, it is greatly affected by the level of operation technology and bone window. Shreve et al. (2019) and Ajčević et al. (2021) highlighted the value of quantitative EEG as a possible complementary tool in the evaluation of stroke severity and its potential role in acute ischemic stroke monitoring. Reid et al. (2010) have shown that outcome prediction was not significantly improved with CT-derived radiological variables or more complex clinical variables. Ajevi et al. (2020) highlighted the importance of CT perfusion (CTP) for decision-making and prediction in the hyperacute phase of wake-up stroke (WUS). These studies have certain practicability, but their sensitivity is limited.

Magnetic resonance imaging (MRI) is the preferred non-invasive method for stroke screening (Nakagomi et al., 2019). Traditional MRI equipment is impossible to carry in a standard mobile vehicle and rural grass-roots hospitals lack the funds to purchase MRI equipment. Hence, a specifically designed mobile MRI system was designed which can diagnose stroke and related changes to the brain in resource deficient environments without the strict positioning requirements and high cost of traditional large scanners (Nakagomi et al., 2019), and it can be used to screen stroke in remote areas. The benefits of mobile MRI system include avoiding costly remodels to install diagnostic equipment in facility, providing diagnostic services to areas where it may be a scarcity and reducing capital expenditures due to possible budgetary constraints. However, the disadvantage of the mobile MRI system is that the diagnostic results of the images may also be partially different from those of the medium-high field MRI device due to the use of a 0.3 T on-board MRI device and parameter settings. To our knowledge, there is no report about the application of mobile MRI system in stroke screening at present. Thus, this study aimed to evaluate a predictive model for stroke that combined conventional risk factors with mobile MRI.

## Methods

### Study design and participants

This was a prospective case-control study enrolled residents over 40 years old in remote rural areas of northern China between May 2019 and May 2020. Health examination population without contraindications to MRI examination were eligible. The exclusion criteria included (1) contraindications to MRI examination such as the presence of metal implants, pacemakers, or artificial heart valves and claustrophobia, (2) insufficient number of images or poor images taken during the MRI, (3) a lack of baseline data, (4) a history of severe traumatic, toxic, or infectious brain injury, brain tumors, dementia, severe ischemic stroke, or hemorrhagic stroke, and (5) loss of follow-up or death during follow-up (Supplementary Figure 1).

This protocol was approved by the ethics committee of Baotou Central Hospital [No. KYLL2019 (Lun) 021]. All subjects signed written informed consent before examination.

### Data collection

On the day of physical examination, a uniformly trained medical staff interviewed each subject face-to-face to obtain their medical data, including demographic information (age and gender), behavior and lifestyle habits (smoking, drinking, and physical exercise), disease history (hypertension, diabetes, cardiovascular and cerebrovascular diseases, and lipid metabolism abnormality), and medication prescribed in the past 3 months. Specially trained doctors and nurses measured the height, weight, waist circumference, and blood pressure of each patient. The subjects were instructed to fast before anterior elbow venous blood collection, which was sub–packed in anticoagulant free, EDTA, and heparin anticoagulant tubes and centrifuged. The professional examiner used an automatic biochemical analyzer to measure fasting blood glucose, glycosylated hemoglobin, homocysteine (HCY), triglyceride, total cholesterol, low-density lipoprotein, and high-density lipoprotein.

The stroke risk screening assessment was conducted according to the following 8 risk factors where 1 point is given for each positive characteristic. (1) History of hypertension (≥140/90 mmHg) or current prescription for antihypertensive drugs. (2) Diagnosis of a heart abnormality such as atrial fibrillation and/or valvular heart disease. (3) Smoking. (4) Dyslipidemia. (5) Diabetes mellitus. (6) Physical inactivity. (7) Significantly overweight or obese (BMI ≥ 26 kg/m^2^). (8) Family history of stroke. Patients with scores greater than 3 were considered at high risk.

#### Mobile MRI acquisition

An overview of the mobile MRI system used in this study is shown in Supplementary Methods and Supplementary Figure 2 (XiBaoBoWei, China). The mobile on-board magnetic resonance system was carried in a Mercedes Benz Actros2636E5, and was equipped with an on-board generator to supply energy to the whole system. It was equipped with a mini and super functional Vivi X330 0.3T MRI system (XiBao Medical, China). A remote emergency monitoring system was used for 24-h observation, monitoring and telemedicine guidance.

#### MRI parameters

A ViviX330 0.3T mobile vehicle mounted MRI scanner (XiBao Medical, China) was used to image the patient in a supine position, so that the long axis of the human body was consistent with the long axis of the bed surface, and the head was placed in the coil. The coronal, sagittal, and axial localization images were made simultaneously using the fast recommended imaging sequence. Conventional cross-sectional T1WI, T2WI, and FLAIR sequences were performed. The scanning parameters are shown in Supplementary Figure 3.

#### Evaluation criteria for mobile MRI stroke screening

Based on the standards to report vascular changes in neuroimaging (STRIVE) (Wardlaw et al., 2013), patients were evaluated for WMH, lacunar infarcts, EPVs and brain atrophy, and the details are presented in the Supplementary Material. Chronic cortical infarction was also considered as an incidental manifestation of cSVD and a potential contribution to clinical results (Jokinen et al., 2020). However, due to the timeliness of screening, magnetic sensitivity and diffusion weighted imaging were not routinely performed, and microbleeds and recent subcortical infarction were not evaluated. All MRI results were evaluated by two experienced neuroradiologists to determine the existence, location and size of cerebral small vessel lesions. If there were different opinions, they were resolved through discussion between examiners.

An ordinal score of 0–5 was constructed to reflect the total burden of cSVD. Patients were evaluated for the presence of moderate to severe brain atrophy, one or more lacunar infarcts, periventricular WMHs with Fazekas scores (Cedres et al., 2020) of 3 or deep WMHs with Fazekas scores of 2 or 3, moderate extensive (more than 10) EPVs in basal ganglia, and chronic cortical infarction, and any of them will be scored 1 point, respectively. The above cSVD marker scores of each patient are added to calculate the total cSVD score. Therefore, the total score of cSVD ranges from 0 to 5. According to the total cSVD score, each patient was divided into low risk (cSVD total score 0 and 1), medium risk (cSVD total score 2 and 3) and high risk (cSVD total score 4 and 5) groups (Supplementary Figure 4). A representative high-score MRI scan is shown in Supplementary Figure 5.

### Follow up

Follow up was conducted every 12 months by telephone or in person interview. If the subject died or was lost to follow up, the follow up time was the time up to death or loss. The follow up personnel were doctors and researchers who received unified training and used a standard questionnaire. If the subjects were unable to complete the follow up questionnaire due to aphasia, the family members would answer the questionnaire. The total follow up duration was 2 years.

Stroke was definitively diagnosed according to the presence and duration of symptoms and evidence of cerebral infarction shown by MRI. The symptoms of stroke included rapidly developing limb weakness, aphasia, ataxia, hemispatial neglect, and disturbance of consciousness or hemianopsia lasting for more than 24 h, with no obvious cause other than vascular origin. Cerebral infarction on MRI was defined as diffusion weighted imaging with high signal (acute cerebral infarction) or T1 low signal and T2 high signal (subacute cerebral infarction) (Wang et al., 2017). Patients with evidence of stroke during the follow up period were assigned to the stroke group.

### Statistical analysis

The SPSS 26.0 (IBM, Armonk, NY, United States) software was used for statistical analysis. The measurement data are described by mean ± standard deviation (SD) or quartile range, and the number of use cases (n) and percentage (%) of classified data are expressed. The independent samples *t*-test, Mann Whitney *U* non-parametric rank sum test, chi square test or Fisher’s exact test was used for comparison between groups. Multivariate analysis was conducted using a binary logistic regression model, and each independent variable was a factor with *P* < 0.05 from univariate analysis. The results were expressed by corrected odds ratios (OR) and the corresponding 95% confidence interval (CI). The regression coefficient obtained by binary logistic regression was used to calculate the joint index of the index. The receiver operating characteristic curve (ROC) was used to evaluate the diagnostic efficiency of the index, and the sensitivity and specificity of each index were calculated. The area under the curve (AUC) was calculated. Two sided *P* < 0.05 was considered significant difference.

## Results

A total of 1,301 people residing in remote and poor areas in Central and Western Inner Mongolia met the inclusion criteria and received mobile vehicle MRI screening. Among them, 27 subjects were excluded due to loss to follow-up, 2 subjects were excluded due to death, 45 subjects were excluded due to a previous history of ischemic stroke, and 3 subjects were excluded due to an insufficient number of uploaded images or poor image quality. Finally, 1224 cases (Average [SD] age, 59 [SD]; 500 men [40.8%]) were included in this study (Table 1). A total of 56 individuals developed stroke during the study period (stroke group), while the remaining individuals did not (non-stroke group).

**TABLE 1:** Characteristics between the two groups with or without occurrence and outcome.

Characteristic	Non-stroke group	Stroke group	*P*-value
Male, n (%)	470 (40.2)	29 (51.8)	0.095
Age, year	59.00 ± 8.50	64.05 ± 7.27	**<0.001**
BMI, kg/m^2^	25.36 ± 3.71	25.50 ± 3.92	0.747
Waist, cm	88.85 ± 9.91	92.48 ± 12.16	**0.020**
Smoke, n (%)	372 (31.8)	21 (37.5)	0.382
Drink, n (%)	281 (24.1)	16 (28.6)	0.523
Lack of exercise, n (%)	116 (9.9)	10 (17.9)	0.069
Cerebrovascular disease, n (%)	10 (100.0)	0 (0.0)	1.000
Heart disease, n (%)	137 (11.7)	13 (23.2)	**0.015**
Hypertension, n (%)	391 (33.5)	23 (41.4)	0.249
Diabetes, n (%)	135 (11.6)	14 (25.0)	**0.005**
Dyslipidemia, n (%)	159 (13.6)	15 (26.8)	**0.009**
SBP, mmHg	136.22 ± 20.00	144.55 ± 21.79	**0.003**
DBP, mmHg	88.85 ± 9.91	82.73 ± 13.66	0.087
FPG, mmol/L	5.41 ± 1.56	5.63 ± 1.40	0.308
HbA1c, %	5.49 ± 1.17	5.83 ± 1.11	**0.007**
TG, mmol/L	1.72 ± 0.75	2.01 ± 0.96	**0.025**
TC, mmol/L	4.25 ± 0.97	4.50 ± 1.34	0.131
LDL-c, mmol/L	2.28 ± 0.76	2.52 ± 0.87	**0.041**
HDL-c, mmol/L	1.44 ± 0.38	1.36 ± 0.30	0.189
HCY, μmol/L	15.02 ± 11.99	23.54 ± 16.46	**<0.001**

BMI, body mass index; SBP/DBP, systolic/diastolic blood pressure; FPG, fasting plasma glucose; HbA1c, glycosylated hemoglobin; TG, triglyceride; TC, total cholesterol; LDL-c/HDL-c, Low/high density lipoprotein cholesterol; HCY, homocysteine. The bold values mean the data has a significant difference (P < 0.05).

Individuals who developed stroke were significantly older (*P* < 0.001), had significantly higher occurrence of heart disease (*P* = 0.015), diabetes (*P* = 0.005), dyslipidemia (*P* = 0.009), and had significantly increased in waist circumference (*P* = 0.02), systolic blood pressure (SBP) (*P* = 0.003), glycosylated hemoglobin (HbA1c) level (*P* = 0.007), triglyceride (TG) level (*P* = 0.025), low density lipoprotein cholesterol (LDL-c) level (*P* = 0.04), and homocysteine (HCY) level (*P* < 0.001) than patients who did not experience stroke (Table 1 and Supplementary Figure 6).

The patients were stratified into different risk levels based on the national stroke screening and evaluation criteria. Stroke occurred in 10 cases (2.2%) in low-risk group, 16 cases (3.6%) in moderate-risk group and 30 cases (9.1%) in high-risk group. In contrast, as risk assessment based on the mobile MRI screening evaluation criteria, stroke occurred in 8 patients (1.4%) in low-risk group, 10 patients (2.2%) in the medium-risk group, and 40 cases (11.6%) in the high-risk group. Regardless of the risk assessment method, the high-risk groups had significantly more patients who experienced stroke than the low-risk and moderate-risk groups (*P* < 0.05) (Table 2).

**TABLE 2:** Comparison of end point events among different risk groups under national stroke criteria and mobile MRI screening.

	n	Non-stroke group	Stroke group	χ^2^	*P*-value
National criteria screening					
Low risk	448	438 (97.8)	10 (2.2)	22.204	**<0.001**
Moderate risk	447	431 (96.4)	16 (3.6)		
High risk	329	299 (90.9)	30 (9.1)		
Mobile MRI					
Low risk	420	414 (98.6)	6 (1.4)	54.495	**<0.001**
Moderate risk	459	449 (97.8)	10 (2.2)		
High risk	345	305 (88.4)	40 (11.6)		

The bold values mean the data has a significant difference (P < 0.05).

Multivariate logistic regression analysis showed that age (OR = 1.055, 95% CI: 1.017–1.094, *P* = 0.004), HCY (OR = 1.029, 95% CI: 1.012–1.047, *P* = 0.001), and mobile MRI results (OR = 4.539, 95% CI: 1.726–11.939, *P* = 0.002) were independently associated with stroke (Table 3).

**TABLE 3:** Univariate and mutivairate logistic regression analysis.

Variables	Univariate regression	*P*-value	Mutivairate regression	*P*-value
	Odd Ratio, 95% CI		Odd ratio, 95% CI	
Heart disease	1.867, 0.917–3.799	0.085		
Diabetes	1.550, 0.754–3.188	0.233		
Hyperlipidemia	1.188, 0.565–2.497	0.650		
Age	1.052, 1.013–1.094	**0.009**	1.055, 1.017–1.094	0.004
Waist	1.015, 0.984–1.046	0.347		
SDP	1.003, 0.987–1.020	0.692		
HbA1c	1.037, 0.821–1.310	0.759		
TG	1.251, 0.916–1.707	0.159		
LDL-c	1.352, 0.964–1.895	0.080		
HCY	1.033, 1.015–1.051	**<0.001**	1.029, 1.012–1.047	0.001
Mobile MRI	4.611, 1.735–12.254	**0.002**	4.539, 1.726–11.939	0.002
National criteria screening	0.981, 0.356–2.705	0.971	1.850, 0.812–4.214	0.078

SBP, systolic blood pressure; HbA1c, glycosylated hemoglobin; TG, triglyceride; LDL-c, Low density lipoprotein cholesterol; HCY, homocysteine; MRI, magnetic resonance imaging. The bold values mean the data has a significant difference (P < 0.05).

**FIGURE 1:**
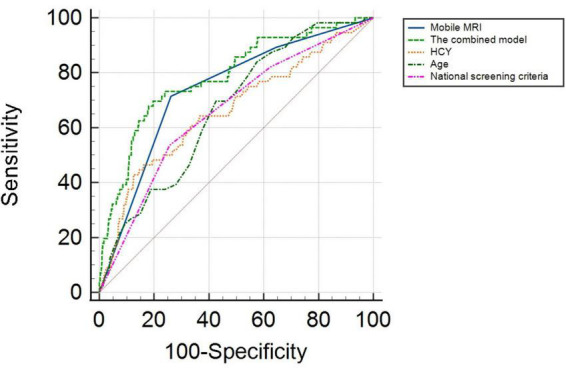
ROC curve of early warning value of different indicators for end events.

ROC analysis showed that the AUC for age was 0.666 (95% CI: 0.602–0.731) with a sensitivity of 83.9%, and specificity of 46.2%. The AUC for HCY was 0.662 (95% CI: 0.582–0.743), and the cutoff value was 24.300 μmol/L, with a sensitivity and specificity of 42.9 and 87.5%, respectively. The AUC of mobile vehicle MRI was 0.738 (95% CI: 0.672–0.803), with a sensitivity of 71.4%, and specificity of 73.9%. The AUC of the combined model (including age, HCY, mobile MRI, and national screening criteria) was 0.786 (95% CI: 0.721–0.851) with a sensitivity and specificity of 69.6 and 80.4%, respectively (Figure 1).

## Discussion

This study showed that the combined model, including age, HCY, national screening criteria, and mobile MRI, had the best predictive capability for stroke in remote areas. These results demonstrate that this predictive model for stroke may allow early intervention for the prevention of cerebrovascular events.

Cerebral small vessel disease is a common cause of stroke. Evidence from observational studies shows that the total cSVD burden is associated with damage to the integrity of the blood-brain barrier and subsequent cognitive impairment, post-stroke depression, and lower post-stroke healthy quality of life (Wang et al., 2021). In this study, WMH, lacunar infarcts, EPVS and cerebral atrophy were used to establish mobile vehicle MRI stroke screening and evaluation criteria for chronic cortical infarction, and its predictive value was verified by 2-year follow-up. The results showed that the occurrence of stroke was significantly greater in the MRI determined high-risk population. Houwei Du et al. (2021) reported that during 2.3 years of follow-up of 1,419 eligible patients, 53 patients developed ischemic stroke, with an end-point event rate of 3.7%. After adjusting for congestive heart failure, hypertension, age >75 years, diabetes, stroke, vascular disease, age 65–75 years, and female scores, the presence of cSVD was independently associated with ischemic stroke.

Some studies have shown that advancing age significantly increases the risk and occurrence of ischemic stroke (Wang et al., 2017). Moreover, the prevalence, morbidity and mortality of ischemic stroke are greater in men than women (Chen et al., 2017). Hypertension is the most important risk factor for stroke. There is a strong, continuous, consistent, and independent correlation between hypertension and stroke (Whelton and Williams, 2018). Additionally, studies have shown that smoking is an important and independent risk factor for ischemic stroke and can increase the relative risk of ischemic stroke by 90% (Rodriguez et al., 2002; Feigin et al., 2005). Diabetes is another independent risk factor for stroke, and diabetes can more than double the risk of stroke (Banerjee et al., 2012). Atrial fibrillation and other types of heart disease may also increase the risk of stroke (Das et al., 2008) and there is a significant correlation between dyslipidemia and stroke (Sun et al., 2019).

Increasing regular daily physical activity can reduce the risk of stroke independently of gender or age (Lobelo et al., 2018). There also exists a hierarchical positive correlation between stroke and obesity, independent of age, lifestyle, or other cardiovascular risk factors (Prospective Studies Collaboration et al., 2009). A large number of studies support the relationship between the increase of plasma HCY levels and atherosclerotic diseases, which can increase the risk of atherosclerotic vascular diseases including stroke by 2–3 times (Das et al., 2008). Nevertheless, the present study showed that heart disease, diabetes, dyslipidemia, age, waist circumference, SBP, HbA1c, TG, LDL, and HCY were all potential risk factors for acute ischemic stroke, and age and HCY were independent risk factors for acute ischemic stroke (OR > 1). These findings were not completely consistent with previous studies (such as gender, obesity, smoking, drinking, lack of exercise, etc.), which may be mainly because of the complexity of the interaction between risk factors, the influence of heredity, race and geography stratification factors, disease progression, guidance of clinical application, small sample size, and short follow-up time.

This study still has the following limitations and deficiencies. First, the sample size was still relatively small owing to the unique target population of this study and imparts a certain selection bias. Second, this study used a visual scoring system of MRI results to represent the total burden of cSVD and cerebrovascular disease. These scales are practical, but their sensitivity is limited, and their predictive value needs to be further validated. Since SVD continuously develops, it may be worthwhile to use a continuous rather than a sequential scale to evaluate the overall lesion load. Computer-generated MRI segmentation can already be used to assess different types of SVD related changes (Jokinen et al., 2020). Finally, the area under the newly established screening standard curve and diagnostic index are still relatively low, so the next step will be to identify new biomarkers for acute ischemic stroke to supplement this model.

In conclusion, this study establishes a simple, safe, non-invasive and accurate early warning model combined with the national screening criteria, mobile MRI, age, and HCY for the first time, which is of great significance for realizing the early detection, early diagnosis and early treatment of ischemic stroke. This study has solved the problems of difficult mobile deployment and use of traditional MRI equipment, lack of MRI equipment in grass-roots hospitals and high MRI examination cost, filled the gap of domestic mobile MRI equipment, marked the application and promotion of large-scale imaging equipment in the field of mobile medicine, and will play a great positive role in remote first aid, joint treatment and virtual surgery of cardiovascular and cerebrovascular diseases. It has very important practical significance for China’s health undertakings.

## Data availability statement

The original contributions presented in the study are included in the article/Supplementary material, further inquiries can be directed to the corresponding author.

## Ethics statement

The studies involving human participants were reviewed and approved by the Baotou Central Hospital [No. KYLL2019 (Lun) 021]. The patients/participants provided their written informed consent to participate in this study.

## Author contributions

KS designed the study and revised the manuscript. ML collected data and wrote the articles. QL consulted literature and wrote the articles. GC designed the study and collected data. NS and MZ wrote MRI diagnostic reports. XL revised the manuscript. All authors approved the final version of the manuscript to be published.
