# Supplementary material for: The value of mobile magnetic resonance imaging in early warning for stroke: A prospective case-control study
Source: Front Neurosci. 2022 Aug 12;16:975217. doi: 10.3389/fnins.2022.975217 (PMC9411978; doi:10.3389/fnins.2022.975217)
Supplement: Supplementary file 1 [file Data_Sheet_1.docx]

**SUPPLEMENTAL MATERIALS**

**Supplemental methods**

**Mobile magnetic resonance imaging system**

ViviX330 magnetic resonance imaging system: it is composed of magnet, two-dimensional electric sickbed, RF system and coil, gradient system and computer system. The magnet is 0.3 ± 0.015T permanent magnet double column open magnet, which adopts self-balancing shimming technology. The magnet shimming type is fourth-order active shimming, the shimming channel is 10 channels, and the magnet weighs 8 tons. The RF power of the RF system is ≥ 2KW, the receiving head coil adopts 8-inch head coil, the specification and model is RFT0599, the number of channels is 4, and the area body is standardized Φ 140 mm ball, single receiving working mode. Gradient system: uniaxial gradient field strength is 12mt / m, uniaxial gradient conversion rate is 40mT/m/ms, gradient coil type is self-shielded coil, minimum layer thickness 2D ≤ 0.5mm, 3D ≤ 0.05mm. The computer system is Windows operating system, with CPU number ≥ 2, memory ≥ 4GB, hard disk capacity ≥ 320GB, network and laser camera interface supporting DICOM 3.0 standard interface.

First aid equipment: the system takes Xibao medical magnetic resonance products as the core, and is equipped with wireless ECG machine, defibrillation and pacing monitor, on-board medical refrigerator, oxygen system, automatic on-board stretcher, cloud video consultation system, network camera, on-board audio and video terminal, high-definition camera, etc. it realizes the integration of image information and audio information and sends it to the remote medical platform, The doctors of the remote emergency monitoring system can remotely guide the rescue work in the ambulance, realize the advance of pre hospital first aid, and greatly shorten the golden treatment time of patients.

**Evaluation criteria for mobile MRI stroke screening**

The lacunae are defined as round or oval, with a diameter of 3-15mm, distributed under the cortex, filled with the same signals as cerebrospinal fluid, and related to old infarction or bleeding in the blood supply area of perforating artery. On T2-FLAIR, it showed central cerebrospinal fluid like low signal, surrounded by high signal ring. It can also show high signal on T2-FLAIR, but it shows cerebrospinal fluid like signal on T1, T2 and other sequences.

White matter hyperintensity (WMH) refers to abnormal signals with different sizes in white matter. It shows high signal on T2 weighted sequence, high signal on T2 weighted liquid attenuation inversion recovery (FLAIR) sequence and equal signal or low signal on T1 weighted sequence. According to Fazekas grading standard [13], the high signal of cerebral white matter was scored: periventricular high signal was 0 = no lesion, 1 = cap or pencil like thin layer lesion, 2 = smooth "halo", 3 = irregular periventricular high signal, extending to deep white matter. The single deep white matter hyperintensity is 0 = no lesion, 1 = punctate lesion, 2 = the lesion begins to converge, and 3 = large-area fusion of the lesion.

Enlarged perivascular space (EPVs) refers to the liquid filled space flowing along the typical trajectory of blood vessels when blood vessels pass through gray matter or white matter. The gap signal intensity on all sequences was similar to that of cerebrospinal fluid. They are linear, round or oval, and their diameter is generally less than 3mm. We only calculated the EPVs of the basal ganglia region, because the EPVs of the basal ganglia is considered to be a marker of csvd [14]. They were scored according to the previously described and validated semi quantitative scoring criteria [15] (from 0 to 4). 0 means no lesions, 1 means < 5 lesions, 2 means 5-10 lesions, 3 means > 10 lesions, but they can still be counted, and 4 means countless.

Brain atrophy is defined as a reduction in brain volume that is not associated with a specific macroscopic focal injury, such as trauma or infarction. Therefore, infarction is not included in this indicator unless explicitly stated. We assessed brain atrophy according to the visual scoring criteria of Pasquier et al. [16]. Mild brain atrophy showed the opening of the surrounding sulcus, moderate atrophy showed the widening of the sulcus, and severe atrophy showed the shallowing of the gyrus. Chronic cortical infarction, usually associated with macrovascular disease, was also included in the analysis as an incidental finding of csvd, defined as necrotic tissue located in the cortex (low signal in T1 and flair sequences) [17].

**References**

[13] Fazekas F,Niederkorn K,Schmidt R,et al.White matter signal abnormalities in normal individuals: correlation with carotid ultrasonography, cerebral blood flow measurements, and cerebrovascular risk factors.[J].Stroke,1988,10:1285-8. doi:10.1161/01.str.19.10.1285.

[14] Zhu YC,Tzourio C,Soumaré A,et al.Severity of dilated Virchow-Robin spaces is associated with age, blood pressure, and MRI markers of small vessel disease: a population-based study.[J].Stroke,2010,11:2483-90. doi:10.1161/STROKEAHA.110.591586.

[15] Doubal FN,MacLullich AM,Ferguson KJ,et al.Enlarged perivascular spaces on MRI are a feature of cerebral small vessel disease.[J].Stroke,2010,3:450-4. doi:10.1161/STROKEAH

A.109.564914.

1. Pasquier F,Leys D,Weerts JG,et al.Inter- and intraobserver reproducibility of cerebral atrophy assessment on MRI scans with hemispheric infarcts.[J].Eur Neurol,1996,5:268-72.do

i:10.1159/000117270.

1. Staals J,Makin SD,Doubal FN,et al.Stroke subtype, vascular risk factors, and total MRI brain small-vessel disease burden.[J].Neurology,2014,14:1228-34. doi:10.1212/WNL.000000

0000000837.

**Supplemental Figures**

**
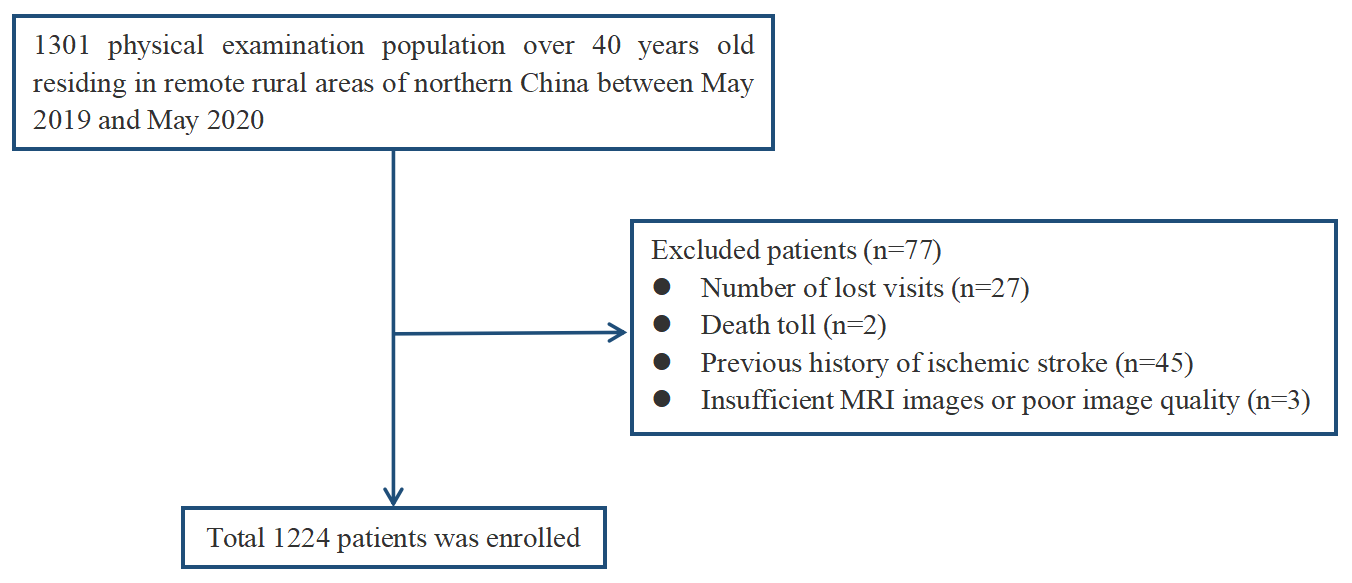
**

**Supplemental Figure 1** An overview of the study workflow.


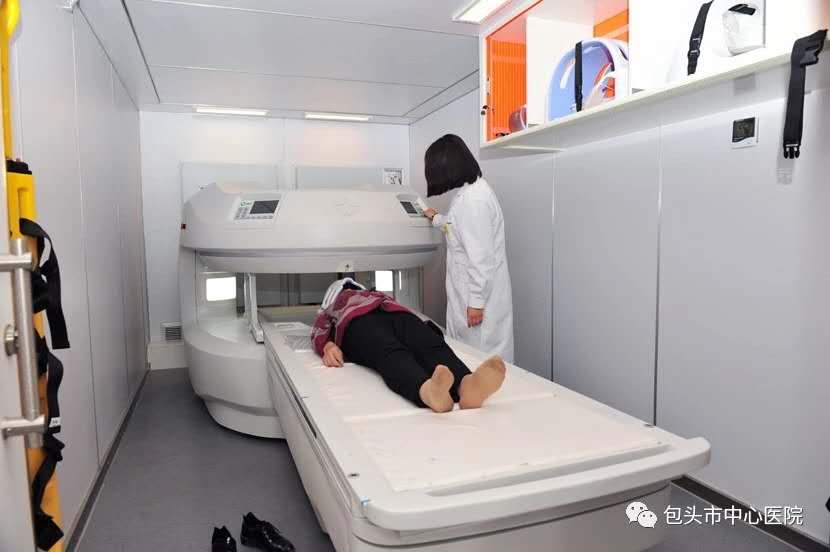
**
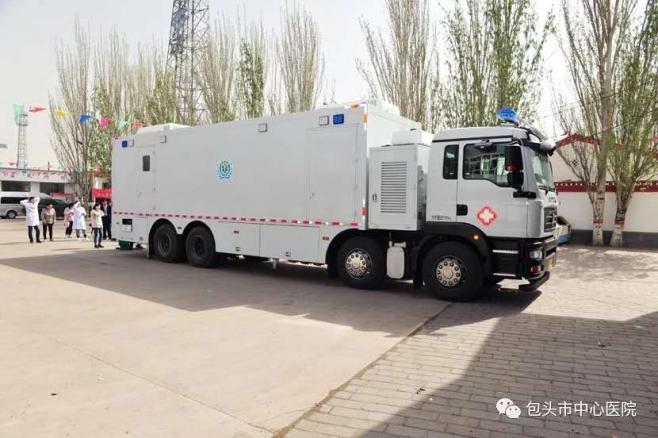
Supplemental Figure 2** Using the first vehicle mounted MRI in China to screen stroke in remote and poor areas.

**Supplemental Figure 3** On-board MRI parameters.

| Parameter, Sequence | T1WI | T2WI | T2 FLAIR |
| --- | --- | --- | --- |
| TR（ms） | 1540.0 | 5700.0 | 9170.0 |
| TE（ms） | 24.01 | 99.35 | 109.35 |
| FOV（mm×mm） | 240×216 | 240×240 | 240×240 |
| Slice thickness（mm） | 7.0 | 7.0 | 7.0 |
| Voxel size（mm^3^） | 1.0×0.8×0.8 | 1.0×0.8×0.8 | 1.0×0.8×0.8 |
| Number of layers | 15 | 15 | 15 |
| TA | 1min31s | 2min28s | 2min36s |

**Supplemental Figure 4** Characteristics and classification of total score of cerebrovascular diseases

| **MRI features** | **Visual evaluation criteria** | **Definition** | **score** | **Typical case diagram** |
| --- | --- | --- | --- | --- |
| lacunar | International consensus definition[18] | Presence of one or more lacunar | 1 | 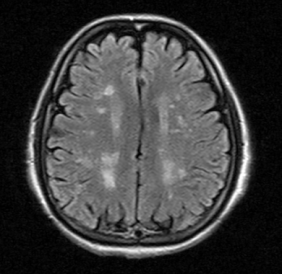 |
| white matter hyperintensity (WMH) | Fazekas grading standard[13] | Periventricular WMH Fazekas score of 3 or deep WMH Fazekas score of 2 or 3 | 1 | 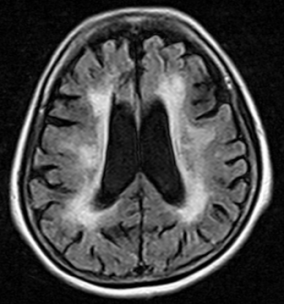 |
| perivascular space enlargement (EPVs) | Semi quantitative evaluation criteria[15] | Moderate extensive (more than 10) EPVs in basal ganglia | 1 | 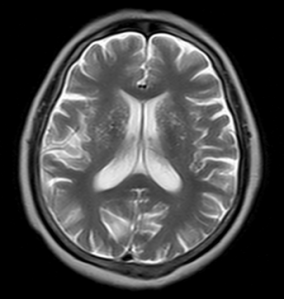 |
| brain atrophy | Scoring criteria of Pasquier et al.[16] | Moderate and severe brain atrophy | 1 | 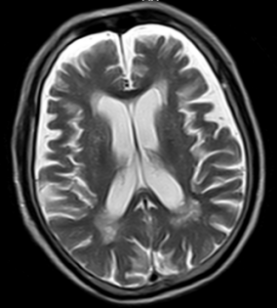 |
| Chronic cortical infarction(Softening stove) | Scoring criteria of Staals et al.[17] | Presence of chronic cortical infarction | 1 | 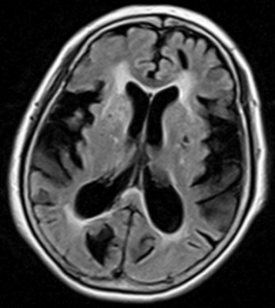 |


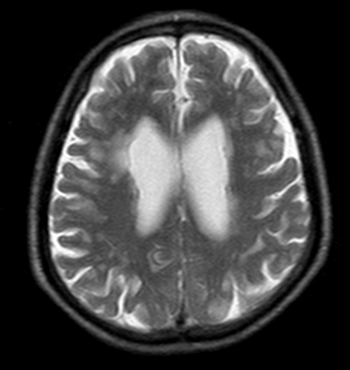

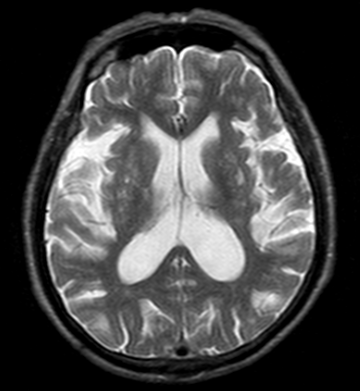

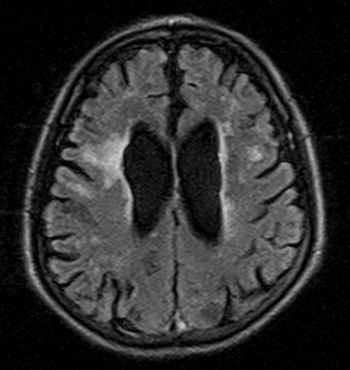

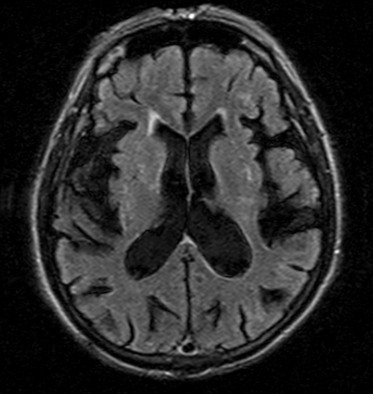


**Supplemental Figure 5** The patient, male, 75 years old, had a history of smoking and hypertension, and no obvious abnormality was found in physical examination and laboratory examination, which was classified as medium risk according to the national stroke screening standard. Brain MRI examination showed multiple lacunae and partial malacia in bilateral frontal parietal lobe, lateral ventricle, basal ganglia, brainstem and left cerebellar hemisphere, periventricular demyelination, widening of vascular space in bilateral basal ganglia and brain atrophy, which were classified as high risk according to vehicle MRI stroke screening criteria. Acute ischemic stroke occurred after a 2-year follow-up.


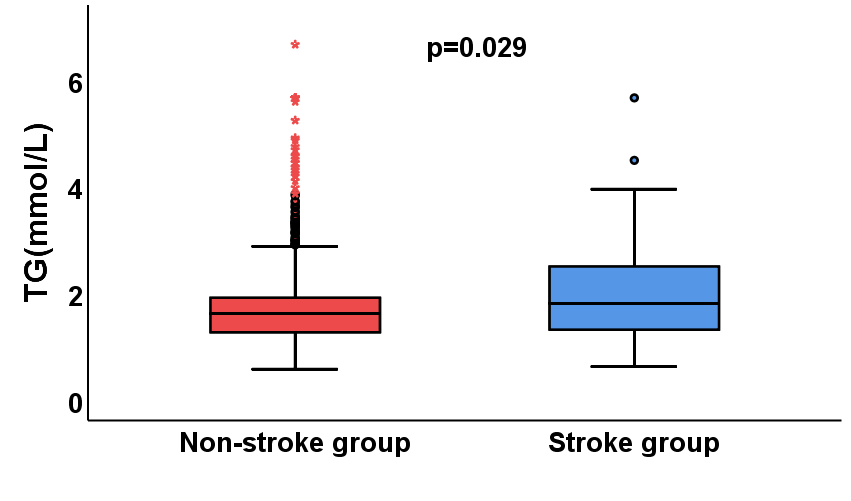

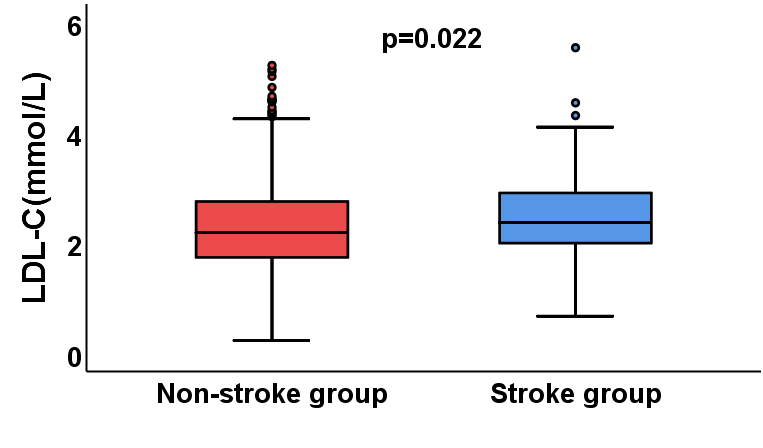


A2

A1


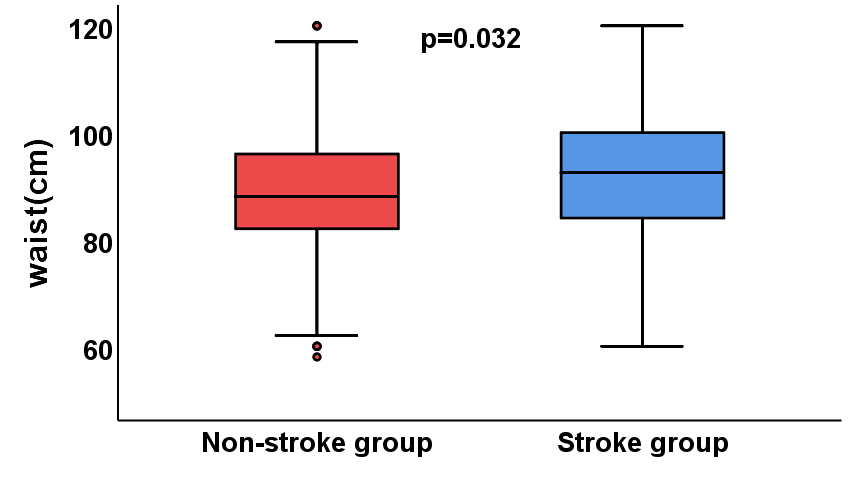

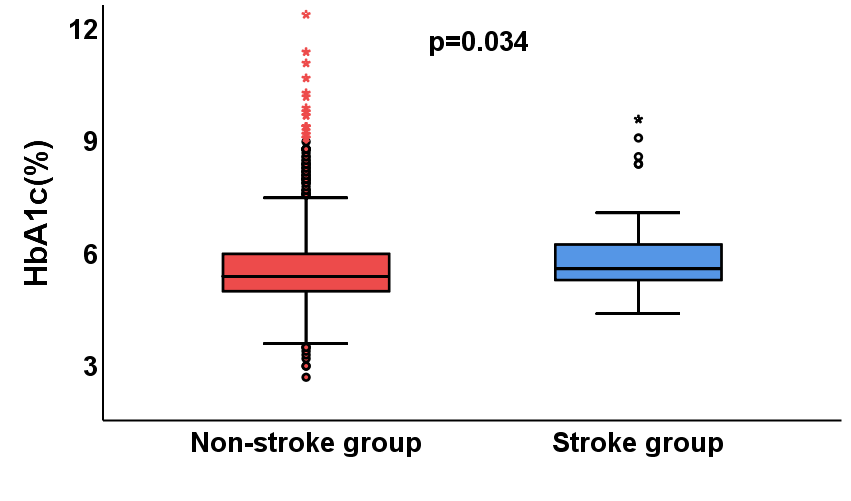


A4

A3


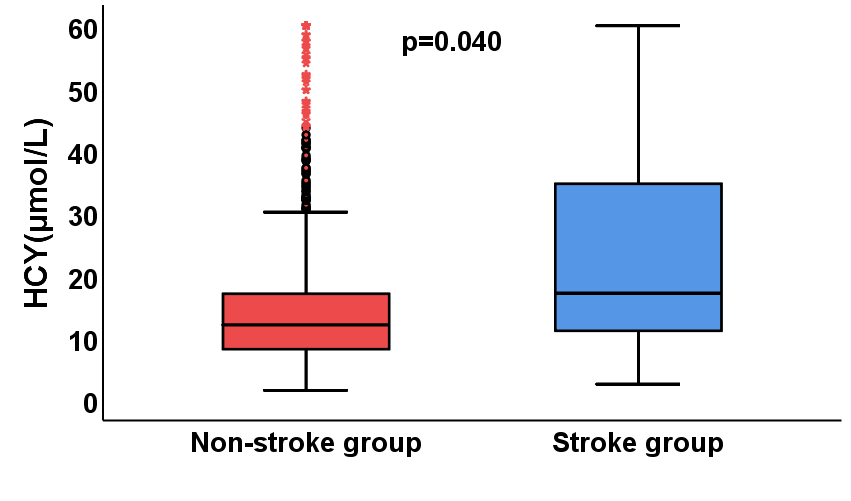

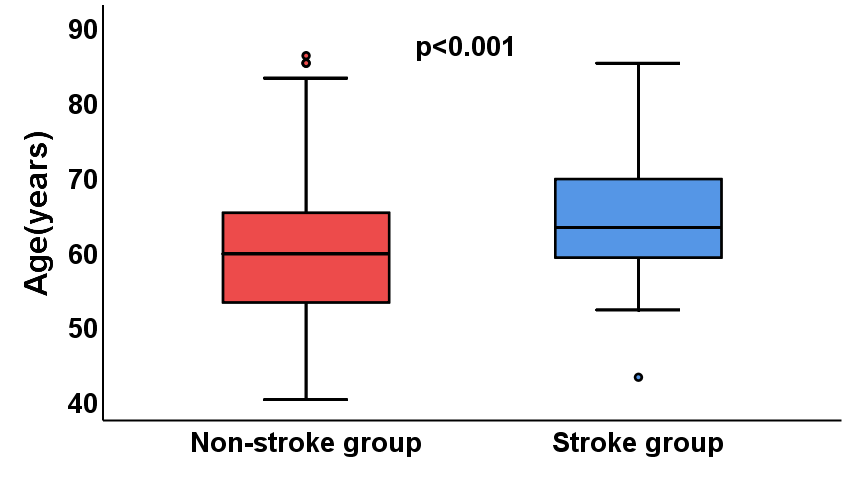


A6

A5


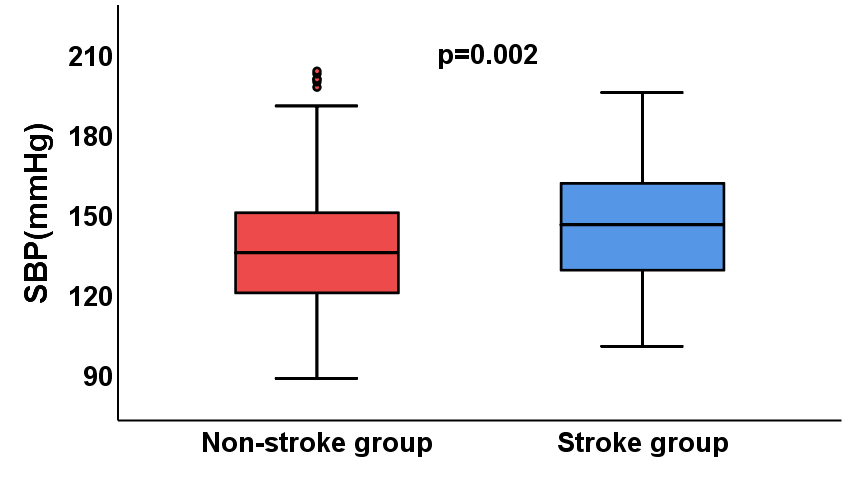


A7

**Supplemental Figure 6** The box diagram (a1-a7) represents the contribution of LDL-C, TG, HbA1c, SBP, waist, age and Hcy to the end event respectively
